# Depression trajectories and their associations with academic performance and sleep patterns among first-year college students

**DOI:** 10.1186/s40359-026-04404-w

**Published:** 2026-05-02

**Authors:** Asal Yunusova, Caitlin Huang, Stephen Price, Kunal Joshi, Janine M. Dutcher, Kirk Warren Brown, Daniella K. Villalba, Michael J. Tumminia, Kasey G. Creswell, Sheldon Cohen, J. David Creswell

**Affiliations:** Department of Psychology, Carnegie Mellon University, 5000 Forbes Ave., Baker Hall, Pittsburgh, PA 15213-3890 USA

**Keywords:** Depression, College students, Clustering, Trajectories, Mental health, Sleep

## Abstract

**Background:**

Depressive symptoms have been on the rise among young adults, with the transition to college, particularly the first year, being a critical period of vulnerability. Despite prior research on depression trajectories in college students, limited longitudinal studies have explored unique depressive symptom trajectory groups among first-year students and their associations with academic achievement (GPA), sleep patterns, and whether sociodemographic factors are associated with certain trajectories.

**Methods:**

This study analyzed a pre-existing dataset that was collected over two waves from a private university (spring semester 2017 and 2018). The final pooled sample resulted in first-year undergraduate students (*N* = 271) who reported on their depressive symptoms (CES-D scale) at the start and end of the semester, signed a release record for their fall and spring term GPA, and provided continuous sleep data across the academic spring term with Fitbits. K-means + + clustering was conducted to form depressive symptom trajectory groups. ANOVAs, Watson-Williams, and Dunnett’s post hoc comparison tests were employed to examine how the resulting trajectory groups were associated with GPA and sleep outcomes (bedtime, waketime, total sleep time, time in bed). Associations between sociodemographic variables and trajectory groups were investigated using chi-square tests.

**Results:**

K-means + + clustering identified four trajectory groups: low-stable (*n* = 109), increasing (*n* = 72), decreasing (*n* = 51), and high-stable depressive symptoms (*n* = 39). The low-stable and decreasing group had a higher spring term GPA (*M* = 3.44 and *M* = 3.39, respectively) compared to the increasing and high-stable groups (*M* = 3.22 and *M* = 3.18, respectively). The low-stable group generally had an earlier wake time and bedtime, greater total sleep time and time in bed, relative to the decreasing and increasing trajectory groups. Gender, ethnicity, international student status, and first-generation student status were not associated with trajectory groups.

**Conclusions:**

Consistent with prior work, there are unique depression trajectory groups among first-year college students that represent stability and change of depressive symptoms over the course of a spring semester. Favorable trajectories (low-stable and decreasing symptoms) are associated with better academic performance and sleep habits.

**Supplementary Information:**

The online version contains supplementary material available at 10.1186/s40359-026-04404-w.

## Introduction

Young adults suffer from high rates of depressive symptomatology, and longitudinal epidemiological studies suggest that these high rates also seem to be rising year after year [1, 2]. The transition to college appears to be a particularly vulnerable period during emerging adulthood, with some indication that the first year of college is a time when depressive symptomatology can spike among adolescents [3–6]. While there have been investigations of depressive symptom trajectories among adolescents and college students [7–14], there is currently a lack of longitudinal studies that investigate the prevalence of unique depressive symptom trajectory groups among first-year students and examine how those groups may differ on grade point average (GPA) and sleep. The current study aims to address these gaps through three aims: (1) to identify unique depressive symptom trajectory groups among first-year students during the spring academic term using a k-means + + clustering approach, (2) to examine whether there are differences in GPA and sleep metrics (collected via Fitbit) between trajectory groups, and (3) to evaluate whether sociodemographic factors might distinguish trajectory group membership.

Group or cluster-based analysis of depression trajectories has been used widely in studying depression trajectories from adolescence to adulthood [7–14] because it helps identify subgroups of depression change (e.g., people who are depressed at time 1 but show reductions in depression at a later timepoint) and depression stability (e.g., people who exhibit consistently high depression over time). Few studies have leveraged these cluster-based analyses for understanding depression trajectory subgroups among young adults going through the first-year college transition [15, 16]. Indeed, students who are in their first or second year exhibit the highest depression [3], and one in three first-year students report experiencing mental health problems [6]. These findings warrant a focus on investigating depressive symptom trajectories among first-year students as they may be particularly at-risk. The current project uses k-means + + clustering– a grouping method that is comparable to those commonly used in previous trajectory work (e.g., latent profile analysis) [17]. Thus, the first aim of the study is to apply k-means + + clustering towards a dataset of first-year undergraduate students to identify unique depressive symptom trajectories across the academic spring semester. While initial studies have focused on linking clinical factors (e.g., affect, stress) and physical health factors (e.g., chronic health conditions) to depressive symptom trajectories, there have been no studies to our knowledge examining whether depressive symptom trajectory groups are associated with markers of academic achievement, like GPA. This is surprising given that undergraduate GPA is a marker for subsequent post-graduate academic achievement such as doctoral degree completion [18], career related outcomes such as job performance [19], and employability perceptions [20]. Moreover, mental health concerns are consistently linked with negative impacts on undergraduate GPA [21–23]. Diagnosed depression has been linked to a GPA decrease of 0.49 points among university students [22] and greater depressive symptoms have been correlated with lower cumulative GPA [24, 25]. Extending previous cross-sectional research into understanding differences in GPA between trajectory groups can clarify whether consistently low depressive symptoms over the course of a student’s first-year spring semester may be more beneficial for academic achievement, or whether ending the semester with low depressive symptoms drives academic achievement (i.e., decreasing depressive symptoms over the academic term).

Sleep has emerged as a critical health behavior that is independently associated with both depressive symptoms and academic outcomes, underscoring its relevance in studies of student well-being. Our group has previously demonstrated that nightly sleep duration during the academic term prospectively associates with end of term GPA among a pool of first- and second-year college students [26], and other work has related higher GPA with greater total sleep time [27], and earlier bedtimes and wake times [28]. Simultaneously, depression has been linked to increased time in bed in healthy adults [29] and later bedtime in college preparatory and university students [30, 31], though the later relation has been found to disappear when sleep duration is accounted for [30]. Additionally, there are mixed findings when investigating the relation between depression and wake time [30, 31]. Initial depressive symptom trajectory studies among first-year students have found that experiencing sleep problems has been linked to a lower likelihood of belonging to the low-stable depressive symptom trajectory group compared to other depressive symptom trajectory groups (high-stable, moderate-increased, recovery, and rapid-increased) [32]. Across college students generally, those who reported greater sleep duration were more likely to be in the increasing depressive symptom trajectory group compared to the low and stable group [33], and better healthy sleep patterns (composite scores examining individual’s chronotype, sleep duration, snoring, daytime sleepiness, and insomnia) has been found to lower the odds of belonging to an increasing depressive symptom trajectory group among adolescent males [34]. However, these studies use self-report measures of sleep, and objective measurements, such as continuous sleep captured via Fitbits, provide a more continuous and potentially sensitive measure of sleep patterns [35] during the first year of college when it is well known that sleep patterns can become disrupted [36–38]. Given the high rates of chronic sleep deprivation during the spring semester among first-year students [39], evidence that insomnia is associated with a twofold increased risk for depression [40], and evidence that young adults with insomnia lasting at least two weeks are at heightened risk for the onset of major depressive episodes or disorder [41], there is a need to examine how understudied sleep factors such as total sleep time, time in bed, wake time, and bedtime are associated with depressive symptom trajectories. Thus, a second aim of the current study is to test whether the resulting trajectory groups from aim 1 are associated with monthly sleep patterns (wake time, bedtime, total sleep time, time in bed) using continuous sleep data captured by Fitbits. Because K-means clustering is an exploratory method that identifies the optimal number of clusters based on the data, there were no a priori hypotheses about the number or characteristics of the trajectories that would result.

Furthermore, some initial studies suggest that sociodemographic factors such as gender and ethnicity [42] may distinguish depression trajectory groups. Females may be at greater risk for unfavorable depression trajectories due to factors such as physiological sex differences and a greater tendency to internalize negative emotions [3]. Additionally, ethnically minoritized students may face heightened risk due to experiences of discrimination, stigma, underrepresentation on campus, and a diminished sense of belonging [43, 44]. International students also encounter unique academic, sociocultural, and psychological challenges, including language barriers, cultural adjustment, and social exclusion, that may contribute to the onset or worsening of depressive symptoms [45, 46]. Similarly, first-generation college students often face stressors such as financial responsibilities to their families, feelings of achievement guilt, and difficulties with campus integration [47, 48]. Thus, a final aim of this study is to test whether gender, ethnicity, international student status, and first-generation college student status are associated with trajectory group membership.

## Methods

### Participants

Participants were first-year undergraduate students attending a private university located in the Middle Atlantic Region in the United States. Data were originally collected through two phases across two years and approved by Carnegie Mellon University’s Institutional Review Board (phase 1: Spring 2017, IRB approval: STUDY2016_00000421; phase 2: Spring 2018, IRB approval: STUDY2017_00000380). Data collection focused on the spring semester, because the goal was to follow first-year students across a full semester, therefore, the fall semester was used to recruit incoming students. And to stay consistent with the first wave of data collection, phase 2 also focused on the spring semester. For both phases, students were recruited using email lists, campus research websites, the campus psychology participant pool website, and physical flyers that were posted throughout the campus. Both phases included participants who were full-time first-year undergraduate students, between the ages of 18 and 25, owners of a data enabled smartphone, and available from the end of fall semester through the following spring semester to complete study-related activities. Phase 2 also recruited second year students, who were excluded from analyses in the current study. Identifying trajectory groups was exploratory research and thus formal study predictions were not pre-registered.

### Procedure

During phase 1, participants completed a two-part baseline session (Time 1 or T1). Part one was completed in person at the end of the fall semester in 2016. Participants were given an overview of the study, provided written consent, and reported demographic information. Additionally, they were asked to release their academic record and downloaded an application on their phone (AWARE) [49]. At the start of spring semester, participants were emailed a link to complete baseline measurements remotely, including depressive symptoms. Participants returned to the lab to pick up a fitness tracker, the Fitbit Flex 2.0, to wear continuously on their non-dominant wrist throughout the spring semester to capture sleep-related data. During the lab visit, participants downloaded the Fitbit application, created an account with their email address, and synced the Fitbit to the application. Participants were sent home with an information sheet that included a reminder to wear the watch all day every day, along with other tips such as avoiding charging the watch overnight. Throughout the semester the AWARE application also collected data such as participants’ location and sent ecological momentary assessments (EMA) during week 1, 7 and 15. At the end of the spring semester (Time 2 or T2) students were emailed another survey link where they repeated the self-report questionnaires from T1, and returned to the lab to sync their AWARE data, receive their final compensation (up to 225. For both phases, compliance for wearing the Fitbit was not monitored, however students were told that they could keep the Fitbit if they wore it for at least 90% of the time throughout the semester. The current study focuses on the self-report survey data, academic release records, and Fitbit sleep data (see additional works [50–55] related to the parent study).

### Measures

#### Demographics

Participants reported their age, gender, ethnicity, college (school or division within the university), and employment status. First-generation college student status and international student status were obtained from institutional records through the university’s Office of Institutional Research and Analytics (IRA).

#### Self-reported depressive symptoms

Depressive symptoms were measured using the 20-item Center for Epidemiological Studies-Depression (CES-D) scale [56], (phase 1 - T1: α = 0.90, T2: α = 0.90; phase 2 - T1: α = 0.88, T2: α = 0.90). Higher scores represent greater levels of depressive symptoms.

#### Academic achievement

GPA for the fall semester and the spring semester was obtained through the university’s registrar office for students who signed academic release forms. The U.S. university grading system uses a scale from 0.0 to 4.0, where a higher GPA number indicates greater academic achievement.

#### Sleep outcomes

The Fitbit application programming interface provided data for daily sleep episodes. Specifically, Fitbits categorized continuous data into ‘sleep’, ‘awake’, or ‘restless’ minutes using a combination of participants’ heart rate and movement [57]. We followed similar preprocessing procedures as in our prior work [26], briefly we first categorized a sleep episode as a minimum of 20 consecutive minutes of non-awake (sleep and/or restless) minutes that was led and followed by a maximum of 5 ‘awake’ minutes. We then created one main sleep episode per day, defined as the longest sleep episode on a given day starting at noon before the following day at noon. We operationalized bedtime as the start time of the main sleep episode and wake time as the end time of the main sleep episode. This difference between wake time and bedtime created our time in bed outcome. Total sleep time (TST) was calculated by subtracting total non-sleep (awake and/or restless) minutes from time in bed. Bedtime and wake time represented minutes after 6:00pm and 4:00am respectively. Given the circular nature of these time-based data, which wrap around a 24-hour cycle, we transformed wake time and bedtime into radians to enable appropriate circular statistical analyses. An additional step we took was to partition the semester data into 4 months, while excluding spring break– phase 1: month 1 (1/17/2017–2/13/2017), month 2 (2/14/2017–3/13/2017), month 3 (3/20/2017–4/16/2017), month 4 (4/17/2017–5/14/2017); phase 2: month 1(1/16/2018–2/12/2018), month 2 (2/13/2018–3/12/2018), month 3 (3/19/2018–4/15/2018), and month 4 (4/16/2018–5/13/2018). Lastly, we only included sleep data from participants who had at least 20% of all possible sleep episodes per month (at least 5 observations) to ensure sufficient longitudinal sleep data.

### Data analytic plan

Phase 1 and phase 2 originally had 188 and 280 participants enrolled, respectively. We filtered the dataset to exclude non-first-year students (phase 2: *n* = 138), dropouts (phase 1: *n* = 28, phase 2: *n* = 20), those with missing GPA (phase 1: *n* = 2, phase 2: *n* = 7), and depressive symptom composite scores (phase 1: *n* = 2). The resulting datasets were pooled together before conducting descriptive statistics on demographic variables of interest and normality tests on our main outcomes of interest. Prior to clustering, the optimal number of clusters (*k*) was determined by using the elbow method [58] and silhouette method [59]. We conducted a k-means + + algorithm on the merged dataset to identify clusters of students with distinct depressive symptom trajectories using T1 and T2 depressive symptom scores. K-means + + is considered an improvement to the general k-means algorithm, because after choosing the initial cluster centers at random, it “weighs the data points according to their squared distance squared from the closest center already chosen” [60]. Furthermore, k-means clustering methods are computationally efficient and provide the capability to explore what the optimal number of clusters are in the dataset [61]. This provides more flexibility in pre-determining the number of groups or trajectories that best describe the data, while still taking empirical evidence into account.

After identifying the clusters, or trajectory groups, we ran one-way ANOVAs to examine whether membership in these groups was associated with GPA and continuous monthly sleep outcomes (total sleep time and time in bed). If an association was detected, a Dunnett’s post-hoc comparison was conducted where the low symptom trajectory group was set as the reference group. The underlying motivation for this was based on our interest in understanding how academic achievement and sleep exhibited by the low symptom trajectory group, compared to the other trajectory groups. For circular outcomes (bedtime and wake time expressed in radians), we conducted Watson-Williams tests to examine overall group differences [62, 63]. Significant effects were followed by pairwise Watson-William comparison tests with Bonferroni corrections. This analytic approach has been applied in prior sleep research involving circular data [64–66]. Finally, we ran Chi-square tests to determine whether there were associations between sociodemographic characteristics and trajectory groups. If an association was detected, a multinomial logistic regression was conducted to estimate the odds of distinct trajectory membership using significant sociodemographic variables. Data cleaning and analyses were conducted using Python [67], R [68], and RStudio [69].

## Results

### Sample characteristics

The final pooled sample^1^ resulted in a total of *N* = 271 students (phase 1: *n* = 156; phase 2: *n* = 115). The majority of participants were 18 years old (*M* = 18.18, *SD* = 0.46) (1 participant was missing a response for their age) and identified as female (57.56%), male (41.70%), or nonbinary (0.74%). Participants reported their ethnicity as Asian (54.61%), White/Caucasian (26.94%), Latino/a (4.80%), Black/African American (4.43%), or multiethnic or other (9.23%). This reflects the demographic composition of students enrolled at the institution where data was collected in 2017 and 2018. See Tables 1 and 2 for more details on participant characteristics.

**Table 1:** Participant demographics

Variable			Frequency	Percentage
Work status	Not working		222	81.92%
	Working part time		49	18.08%
College	Engineering		83	30.63%
	Humanities and Social Sciences		44	16.24%
	Science		40	14.76%
	Computer Science		34	12.55%
	Fine Arts		33	12.18%
	Business		27	9.96%
	Multiple colleges		10	3.69%

**Table 2:** Participant ethnicity

Ethnicity	Frequency
Asian	148
East Asian	104
South Asian	36
Southeast Asian	4
East Asian, South Asian	2
Korean	1
East Asian, Indian	1
White/Caucasian	73
Black/African American	12
Latino/a	13
Multiethnic or other	25
White/Caucasian, Latino/a	7
White/Caucasian, East Asian	7
East Asian, Pacific Islander	3
White/Caucasian, Black/African American	1
White/Caucasian, Middle Eastern	1
White/Caucasian, Greek, and Indian	1
White/Caucasian, South Asian	1
Black/African American, South Asian	1
Latino/a, Native American/American Indian	1
African and Middle Eastern	1
Pacific Islander	1

Participants were able to select more than one ethnicity from the choices presented (White/Caucasian, Black/African American, Latino/a, East Asian, South Asian, Pacific Islander, Native American/American Indian, and Other, where they were able to insert a text response). Individual responses were grouped into broader categories to increase cell sizes for χ² analyses

### Clustering results

**Fig. 1:**
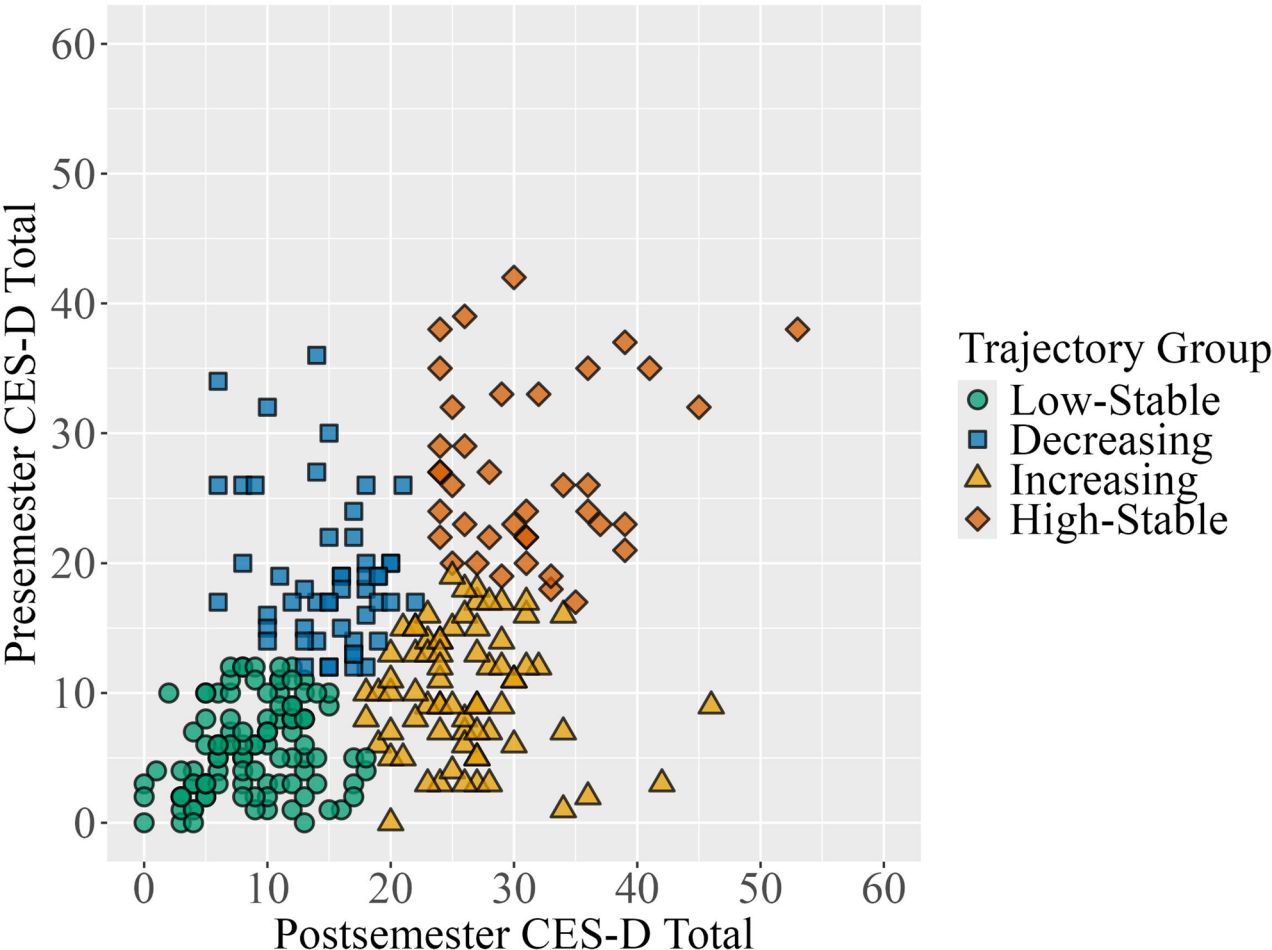
Depressive symptom trajectories using k-means ++

Both the silhouette and elbow method [58, 59] revealed that the optimal number of clusters was 4, therefore we set the clusters to *k* = 4 when conducting the *k*-means + + clustering algorithm (see Figure S1 and S2 in Supplementary Materials). The four clusters that emerged exhibited (1) low depressive symptoms at both the beginning (*M* = 5.73, *SD =* 3.47) and end of the semester (*M* = 8.83, *SD* = 4.20) (*n* = 109), (2) low depressive symptoms at the beginning (*M* = 10.11, *SD* = 4.75) and high symptoms at the end (*M* = 26.00, *SD* = 5.07) (*n* = 72), (3) high depressive symptoms at the beginning (*M* = 19.10, *SD* = 5.84) and low symptoms at the end (*M* = 14.84, *SD* = 4.07) (*n* = 51), and (4) high depressive symptoms at both the beginning (*M* = 26.97, *SD* = 6.73) and end of the semester (*M* = 31.13, *SD* = 6.66) (*n* = 39) (see Fig. 1). These clusters were labeled as the (1) low-stable, (2) increasing, (3) decreasing, and (4) high-stable trajectory groups, respectively. The low-stable trajectory group had the highest number of students (40.22%), followed by the increasing (26.57%), decreasing (18.82%), and high-stable trajectory (14.39%).

### Main analyses

#### Academic achievement

**Fig. 2:**
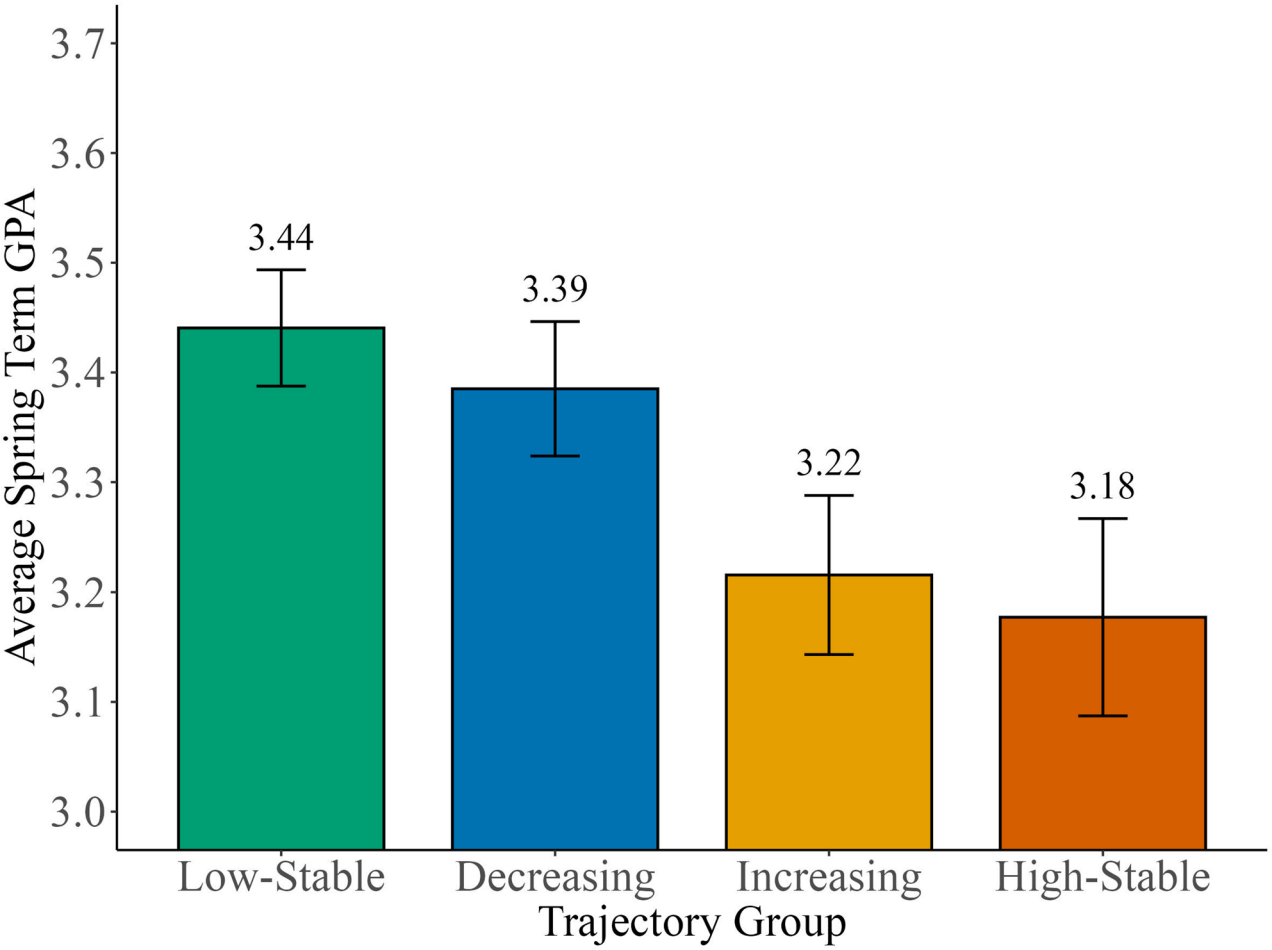
Average spring term GPA by trajectory group. *Note*: Bars represent mean ± SE. The y-axis starts at 3.0. Outliers were included in the main analyses (skewness = -1.09, kurtosis = 4.08). A sensitivity analysis was also conducted, such that outliers detected by the interquartile range (IQR) were winsorized to the next highest GPA (skewness = -0.81, kurtosis = 3.00). Results remained consistent with the original findings

Prior to examining spring term GPA, we examined whether trajectory groups were associated with fall term GPA to understand whether students in these groups were heading into the spring semester with similar academic achievement. A one-way ANOVA revealed there was no difference in previous academic (fall) term GPA between the four groups (*F*_(3, 267)_ = 1.50, *p* = 0.22). However, trajectory membership was significantly associated with spring term GPA (*F*_(3, 267)_ = 3.64, *p* = 0.01). As shown in Fig. 2, mean spring term GPA was higher in the low-stable (*M* = 3.44, *SE* = 0.05) and decreasing trajectory groups (*M* = 3.39, *SE* = 0.06) than in the increasing (*M* = 3.22, *SE* = 0.07), and high-stable groups (*M* = 3.18, *SE* = 0.09). Post hoc Dunnett tests indicated that the low-stable group had a significantly higher GPA than the increasing and high-stable groups but did not differ significantly from the decreasing group (see Tables 3 and 4).

**Table 3:** ANOVA and Watson-Williams results comparing GPA and sleep across trajectory groups

Time	Variable	Low-stable	Decreasing	Increasing	High-stable	*F value*	*p*
		Mean (SD)	Mean (SD)	Mean (SD)	Mean (SD)		
Fall	GPA	3.45 (0.52)	3.36 (0.51)	3.34 (0.65)	3.24 (0.56)	1.50	0.22
Spring	GPA	3.44 (0.55)	3.39 (0.44)	3.22 (0.62)	3.18 (0.56)	3.64	0.01*
Month 1	Wake time	08:56 am (1H 49M)	09:01 am (1H 51M)	09:16 am (1H 55M)	08:56 am (2H 10M)	9.59	<.001***
	Bedtime	01:47 am (1H 50M)	02:18 am (1H 53M)	02:18 am (1H 53M)	01:54 am (2H 20M)	29.44	<.001***
	Total sleep time	6H 44M (1H 38M)	6H 21M (1H 36M)	6H 33M (1H 46M)	6H 36M (1H 43M)	12.96	<.001***
	Time in bed	7H 3M (1H 39M)	6H 38M (1H 41M)	6H 51M (1H 49M)	6H 54M (1H 45M)	14.47	<.001***
Month 2	Wake time	09:08 am (2H 01M)	09:13 am (2H 08M)	09:33 am (2H 12M)	08:56 am (2H 36M)	13.02	<.001***
	Bedtime	02:05 am (2H 08M)	02:28 am (1H 59M)	02:36 am (2H 13M)	01:59 am (2H 40M)	19.84	<.001***
	Total sleep time	6H 37M (1H 50M)	6H 23M (1H 44M)	6H 28M(1H 56M)	6H 28M (1H 52M)	3.81	0.01*
	Time in bed	6H 55M (1H 53M)	6H 41M (1H 50M)	6H 47M (2H 0M)	6H 44M (1H 55M)	3.99	0.01**
Month 3	Wake time	09:03 am (2H 7M)	09:17 am (2H 8M)	09:25 am (2H 27M)	08:59 am (2H 31M)	6.84	<.001***
	Bedtime	02:03 am (2H 12M)	02:37 am (2H 9M)	02:34 am (2H 26M)	02:04 am (2H 40M)	18.15	<.001***
	Total sleep time	6H 35M (1H 47M)	6H 16M (1H 39M)	6H 21M (1H 51M)	6H 29M (1H 57M)	7.24	<.001***
	Time in bed	6H 53M (1H 48M)	6H 33M (1H 45M)	6H 39M (1H 55M)	6H 46M (2H 1M)	6.96	<.001***
Month 4	Wake time	9:15 am (2H 21M)	9:21 am (2H 29M)	9:49 am (2H 27M)	8:47 am (2H 47M)	16.89	<.001***
	Bedtime	02:05 am (2H 20M)	02:30 am (2H 28M)	02:41 am (2H 31M)	01:42 am (2H 46M)	18.97	<.001***
	Total sleep time	6H 38M (1H 52M)	6H 16M (1H 54M)	6H 34M (1H 57M)	6H 30M (2H 1M)	6.59	<.001***
	Time in bed	6H 59M (1H 54M)	6H 35M (1H 59M)	6H 52M (2H 2M)	6H 46M (2H 5M)	6.91	<.001***

*p < .05., **p < .01., ***p < .001

**Table 4:** Post-hoc multiple comparisons of GPA and sleep

Time	Variable	Decreasing vs. Low-stable			Increasing vs. Low-stable			High-stable vs. Low-stable		
		Mean Diff.	Sig.	CI	Mean Diff.	Sig.	CI	MeanDiff.	Sig.	CI
Spring	GPA	-0.06 (0.09)	0.90	-0.28, 0.17	-0.22 (0.08)	0.02*	-0.43,-0.02	-0.26 (0.10)	0.03*	-0.51, -0.02
Month 1	Wake time	–	0.60	–	–	<.001***	–	–	1.00	–
	Bedtime	–	<.001***	–	–	<.001***	–	–	0.60	–
	Total sleep Time	-22.68 (3.67)	<.001***	-31.38,-13.98	-10.59 (3.55)	0.01**	-19.01,-2.17	-7.92 (4.52)	0.21	-18.65, 2.82
	Time in bed	-24.63 (3.77)	<.001***	-33.57,-15.69	-11.61 (3.65)	.004**	-20.26,-2.95	-9.36 (4.65)	0.12	-20.39, 1.68
Month 2	Wake time	–	0.85	–	–	<.001***	–	–	0.16	–
	Bedtime	–	<.001***	–	–	<.001***	–	–	1.00	–
	Total sleep time	-13.56 (4.23)	0.004**	-23.61, -3.52	-8.59 (4.16)	0.11	-18.46, 1.28	-8.58 (5.25)	0.26	-21.05, 3.89
	Time in bed	-14.26 (4.38)	0.003**	-24.66,-3.86	-8.23 (4.31)	0.15	-18.45, 1.99	-10.67 (5.44)	0.14	-23.58, 2.25
Month 3	Wake time	–	0.04*	–	–	<.001***	–	–	1.00	–
	Bedtime	–	<.001***	–	–	<.001***	–	–	1.00	–
	Total sleep time	-18.83 (4.40)	<.001***	-29.26,-8.40	-13.99 (4.38)	0.004**	-24.37,-3.60	-5.20 (5.48)	0.69	-18.21, 7.81
	Time in bed	-19.47 (4.53)	<.001***	-30.22,-8.71	-13.72 (4.51)	0.01**	-24.43,-3.02	-7.02 (5.65)	0.49	-20.42, 6.38
Month 4	Wake time	–	1.00	–	–	<.001***	–	–	0.003**	–
	Bedtime	–	<.001***	–	–	<.001***	–	–	0.02*	–
	Total sleep Time	-22.14 (5.08)	<.001***	-34.21, -10.08	-4.29 (5.16)	0.77	-16.55, 7.98	-8.95 (6.54)	0.41	-24.49, 6.59
	Time in bed	-23.53 (5.26)	<.001***	-36.01, -11.06	-6.28 (5.34)	0.54	-18.97, 6.41	-12.25 (6.77)	0.19	-28.32, 3.82

Mean differences for linear sleep outcomes are reported in minutes along with standard error. Bolded text indicates *p*-values below the .05 significance threshold

*p < .05., **p < .01., ***p < .001

#### Sleep outcomes

**Fig. 3:**
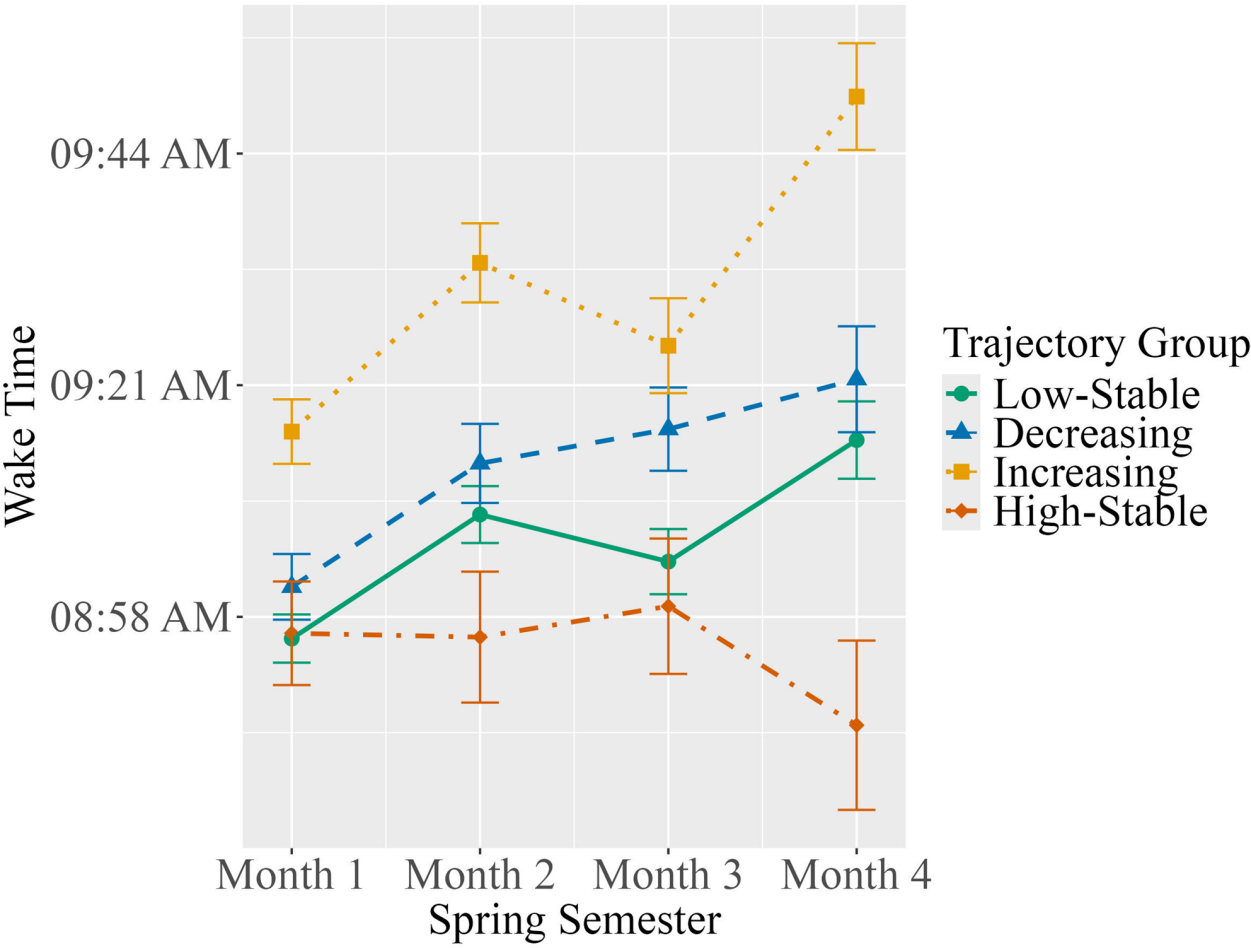
Monthly wake time average by trajectory group

**Fig. 4:**
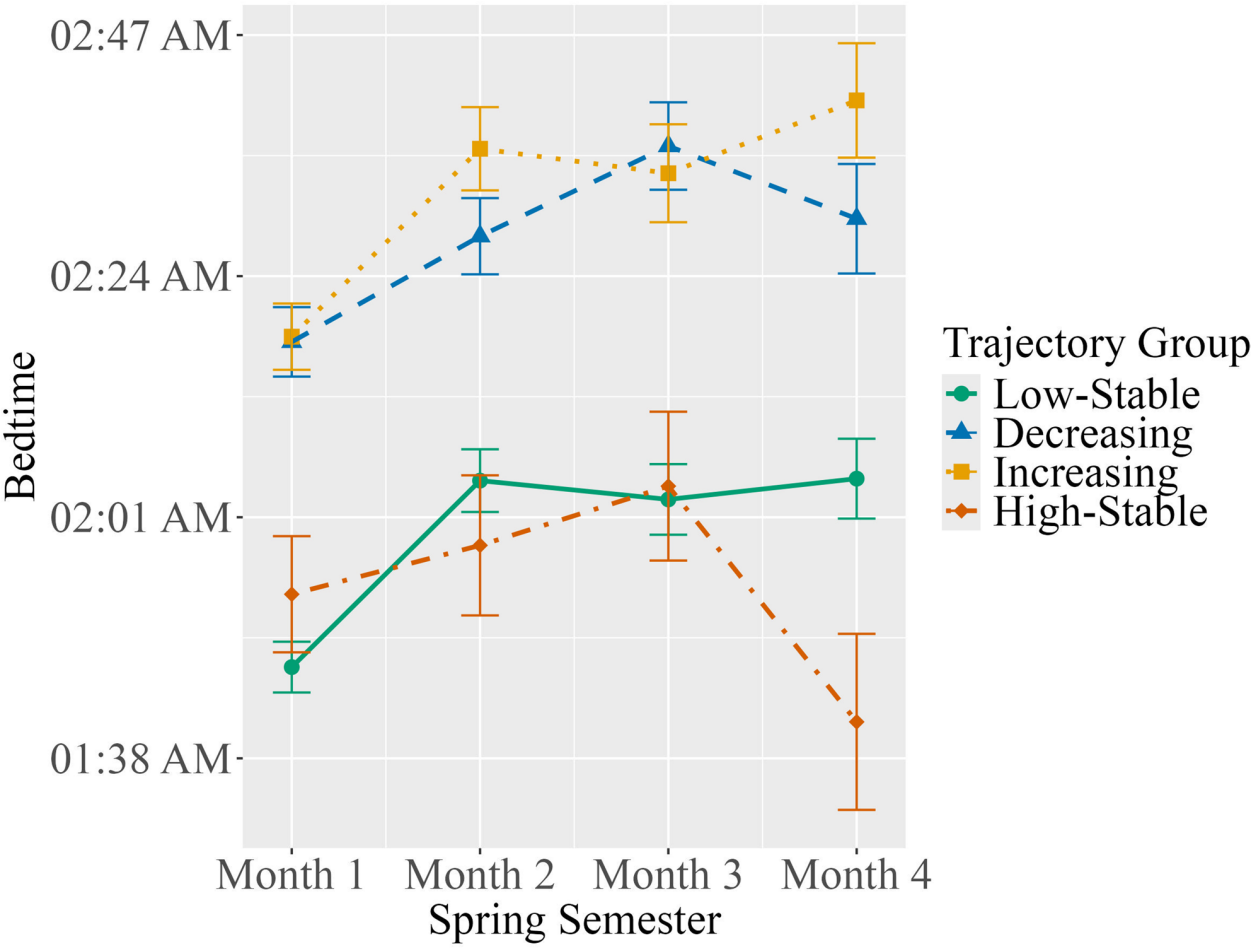
Monthly bedtime average by trajectory group

**Fig. 5:**
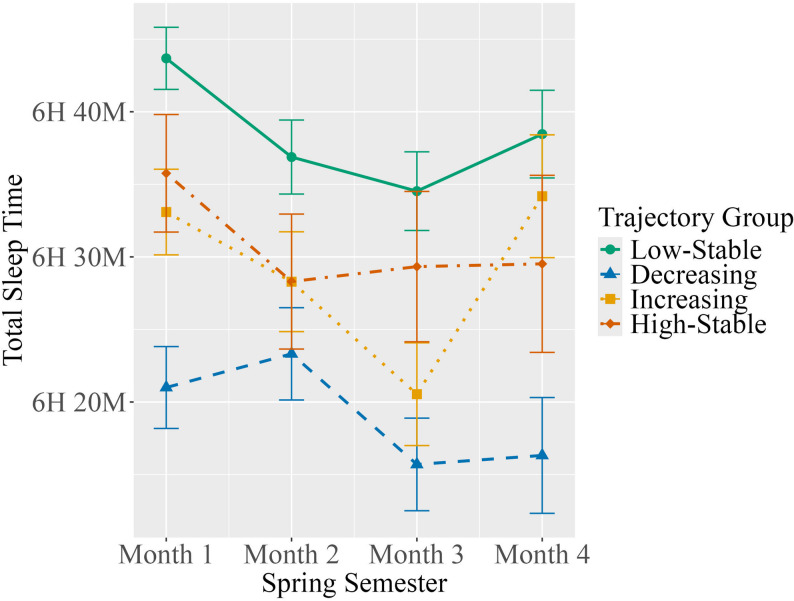
Monthly total sleep time average by trajectory group

**Fig. 6:**
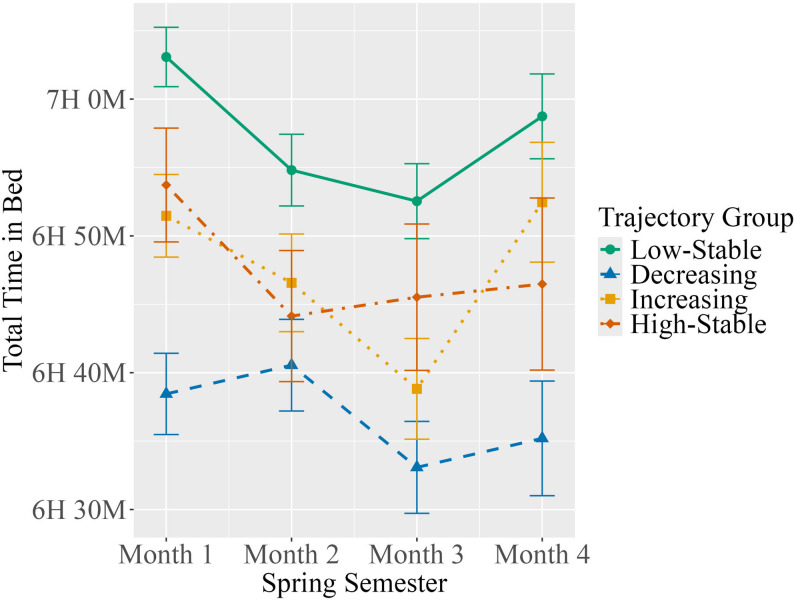
Monthly time in bed average by trajectory group

Overall, trajectory membership was associated with all sleep outcomes across the four months^2^. The greatest differences were observed when comparing the low-stable group to the increasing and decreasing trajectory groups. Generally, the low-stable trajectory group exhibited an earlier wake time and bedtime, and greater total sleep time and time in bed. Only two differences arose between the low-stable and high-stable group – the high-stable group had an earlier bedtime and waketime during the last month only. See Tables 3 and 4; Figs. 3, 4, 5 and 6 for complete statistical reporting and group comparisons. Additional descriptive results are provided in the Supplementary Materials, including sample sizes across clusters and months, (Table S1), distributional properties for linear and circular sleep variables, (Table S2-S4), and proportions of sleep data falling within specific time windows (Table S5).

### Do sociodemographic factors distinguish trajectory groups?

Chi-square and Fisher tests were conducted to examine whether there were group differences for the sociodemographic variables of interest. There was no significant relationship between gender (*X*^*2*^ (3) = 4.93, *p* = 0.18), international student status (*p* = 0.25), first-generation student status (*p* = 0.77), or ethnicity (*p* = 0.46) and trajectory group membership (see Table 5).

**Table 5:** Contingency table for sociodemographic variables with Chi-square and Fisher’s test

Group	Low-stable	Decreasing	Increasing	High-stable	Fisher’s Exact Test or χ^2^
Gender					χ^2^ (3) = 4.93, *p* = 0.18
Female	57	36	40	23	
Non-female	52	15	32	16	
International					*p* = 0.25
Yes	10	4	8	8	
No	99	47	64	31	
First-generation					*p* = 0.77
Yes	11	7	6	3	
No	98	44	66	36	
Ethnicity					*p* = 0.46
White/Caucasian	38	10	16	9	
Black/African American	5	1	3	3	
Asian	50	32	43	23	
Latino/a	6	4	3	0	
Multiethnic or Other	10	4	7	4	
Major					χ^2^ (3) = 1.41, *p* = 0.70
STEM	70	31	40	23	
Non-STEM	39	20	32	16	

Non-binary students (*n* = 2) were grouped with male students to create the non-female category for gender. STEM included students who belonged to the school of engineering, computer science, and science, while non-STEM included fine arts, social sciences, and business. Those who chose multiple colleges were categorized into STEM if at least one of their colleges was engineering, computer science, or science. Fisher’s exact test was conducted in place of Chi-square tests for variables that had small sample sizes in categories. We also conducted a Cramer’s V test to examine the strength of the association between gender and trajectory groups, and found a weak association, Cramer’s V = 0.13

## Discussion

To our knowledge, this is the first study to identify unique depressive symptom trajectory groups among first-year students in the United States, who are at risk for experiencing depression compared to other college years. Using a novel grouping approach, k-means + + clustering, the majority of students were grouped into the low-stable trajectory (40.22%), followed by the increasing (26.57%), decreasing (18.82%), and high-stable trajectory group (14.39%). The trajectories exhibited by these four groups are consistent with prior trajectory work among college students [15, 16, 32–34, 71, 72], but this study extends this previous work in important ways. First, comparing our findings with emerging trajectory research conducted among students outside of the U.S. suggests that there are proportional differences between countries. Q. Liu et al. [16] followed Chinese freshmen across the first semester and identified three groups (‘normal’, 73.1%, ‘depression risk’, 20.7%, and ‘deteriorating’, 6.1%), while Zhou et al. [15] followed Chinese freshmen across the first year and identified three groups (‘moderate’, 83.73%, ‘low-stable’, 8.69%, and ‘high-stable’, 7.58%). It is important to consider the environmental and cultural differences existing between these studies and this current work. Still, our study extends prior work by showing what trajectories arise during the spring semester, underscoring that while a large portion of students may be protected against depression in the fall semester as demonstrated in Q. Liu et al.’s [16] study, the proportions across these trajectory groups may change in the spring semester. Additionally, compared to Q. Liu et al.’s [16] latent growth mixture modeling approach, k-means + + clustering was able to detect more nuanced trajectories despite using fewer assessments of depressive symptoms. Comparing our study to the broader trajectory literature among adolescents and adults highlights the need for future work to incorporate more repeat assessments of depressive symptomatology. This approach would help uncover whether there are more nuanced trajectories such as those that are more abrupt (rapidly increasing symptoms) or those that have a relapse in symptoms (start off with high depressive symptoms, decrease, and then increase again). Secondly, this would help obtain a more comprehensive understanding of the transition and adjustment to college across the first year, given that depressive symptoms at the start of the fall semester predict symptoms in the spring semester among first-year students [73]. Additionally, combining this future investigative work with longitudinal psychosocial, biological, and behavioral measures into time series models can reveal possible mechanisms that drive depressive symptom trajectories.

After identifying the trajectory groups, we investigated the relationship between GPA and depressive symptom trajectories. In line with prior work linking high end of term depressive symptoms to poor academic outcomes in non-first-year samples [24, 25], the high-stable and increasing depressive symptom trajectory groups had the lowest spring term GPA. However, in contrast to our findings, Repetto et al. [74] previously found that high school students in the low depressive symptoms trajectory group had a higher GPA compared to a decreasing symptoms trajectory; though the authors pointed out that their results may have been skewed given the smaller range of GPA in their sample. Our findings advance the field by demonstrating that differences in GPA are associated with depressive symptom trajectories within first-year college students as well, and that the low-stable group and decreasing group exhibit higher GPA compared to other trajectories. Aside from potential benefits to GPA, prior work suggests that being in the low depressive symptom trajectory group might protect against burnout, yield a higher salary, and increase the likelihood of graduating [75]. Combined with our findings, this establishes the importance of building foundational depressive symptom trajectory research among first-year college students. While Salmela-Aro et al. [75] show how unwavering these depressive symptom trajectories can be for certain individuals, with negative consequences in adulthood, our findings suggest that the transition period during the first year of college is an important time for mental health and its role in shaping academic achievement. Although we could not establish the direction of the relationship between depressive symptoms and academic achievement or draw causal inferences, the lack of differences in Fall semester GPA suggests that academic performance was initially comparable across groups, with differences emerging only later in the academic year. This pattern is consistent with the possibility that changes in depressive symptoms may precede subsequent differences in academic functioning. However, it is also possible that these processes are bidirectional [76, 77], such that poor academic functioning/performance may influence psychological functioning, or even a third factor, such as sleep, impacting both processes simultaneously [78]. Together, this work calls attention to the importance of implementing interventions; targeting at-risk students at the start of a semester may potentially prevent negative impacts not only on psychological health, but also on academic achievement. Future work is needed to evaluate how these effects in the first-year of college may extend further during the college years and beyond.

In addition to investigating academic achievement, we aimed to extend prior literature examining self-report measures of sleep and depressive symptom trajectories [32, 33], by capturing sleep metrics using Fitbits worn over the course of the spring academic term. Using a more sensitive and continuous measurement of sleep provides a more accurate representation of college students’ sleep behaviors in real time and in a naturalistic setting, increasing the ecological validity of the results. Current sleep medicine guidelines [79] recommend 7–9 h of sleep for young adults ages 18–25, and there is almost an even split among college students who meet or don’t meet these guidelines. According to a national survey, in spring 2025, 41.6% of students slept less than 7 h on weeknights, and more than half of students reported taking 16 to 30 min (28.7%) or more (29.2%) to fall asleep [80]. Generally, compared to the increasing and decreasing groups, the low-stable group was the closest to meeting these guidelines; however their monthly total sleep time average was still below the 7-9-hour recommendation. And surprisingly, the high-stable and low-stable depression trajectory groups shared similar sleep schedules– both groups exhibited similar wake time, bedtime, total sleep time, and time in bed across all but the last month. Given that approximately half of the study sample were enrolled in STEM (Science, Technology, Engineering, and Mathematics)-related colleges, we considered whether a greater proportion of STEM majors in the low-stable and high-stable clusters might have explained their similar sleep patterns, potentially due to comparable course loads or class schedules. However, a follow-up chi-square test indicated no significant association; STEM majors were distributed similarly across clusters (see Table 5). More broadly, considering that participants were drawn from a highly ranked private university, the academic and institutional rigor may contribute to insufficient sleep all around.

Overall these findings contradict prior work that has linked greater depressive symptoms to longer time in bed [29], insufficient or excessive sleep [33, 39, 40, 81, 82], and late bedtime [30] (unless sleep duration is accounted for [31]). Simultaneously, some studies have found no significant association between depressive symptoms and sleep duration [30]. Our findings are consistent with Supartini et al. [30] who reported that 74.5% of students slept fewer than seven hours per night, with a similar distribution of sleep duration among those with and without depressive symptoms. Other research has documented average bedtimes of 12:35 a.m [83]. and 1:54 a.m [84]., highlighting the prevalence of delayed sleep schedules in this population. Given that both the low-stable and high-stable group have insufficient sleep durations, this suggests that there may also be underlying protective behaviors or factors exhibited by the low-stable group that prevents their depressive symptoms from worsening due to a negative sleep schedule. Another key factor to consider is sleep regularity, or the “intraindividual variability in sleep timing” [85], which has been shown to be a more robust predictor of all-cause mortality compared to sleep duration [86]. As for time in bed, although it has been linked to depression within experimental settings [29], it is difficult to draw conclusions because there are no data regarding what participants were doing before falling asleep. It may be the case that students in the low-stable trajectory group have better sleep hygiene, such as avoiding screen time, compared to those in the high-stable trajectory group, allowing for better sleep quality at night [87]. Furthermore, because sleep episodes were defined by allowing up to 5 min of wakefulness at the start and end, the resulting time in bed may not accurately reflect the actual duration participants spent in bed. Overall, the continued use of wearables such as Fitbits or actigraphy devices to capture sleep metrics will ensure more accurate measurements of sleep, and using a standardized method may clarify any mixed findings concerning sleep and depression moving forward. Finally, we aimed to replicate previous work examining the relationship between gender [7, 9, 32, 33] and ethnicity [42] with depressive symptom trajectories and build upon prior work investigating depression among first-generation college students [88–90] and international students [91, 92]. The absence of gender differences in our study may reflect greater mental health knowledge among female students, as prior work shows females report higher mental health knowledge, which is associated with lower risk for depressive symptoms [93]. In contrast to our findings, racial minority status has been linked to higher depressive symptom trajectories from adolescence to early adulthood [42], though racial minority students also report lower rates of psychiatric diagnosis, likely reflecting disparities in mental healthcare access rather than lower symptom burden [94]. Furthermore, evidence comparing depression among first-generation college students and international students is mixed [88–91, 95], though both groups face unique stressors and lower mental healthcare utilization [96, 97]. Notably, a recent review found that most studies report no significant differences in depressive symptoms between first-generation and continuing-generation students, citing several potential explanations, including the buffering effects of resilience [47]. Overall, examining single identities might hide important patterns [88], and our underrepresentation of ethnically minoritized, international, and first-generation students likely limited power to detect sociodemographic differences. Future research should consider the importance of intersecting social identities and structural inequities in healthcare access when identifying students at risk for unfavorable depressive trajectories. Person-level factors (e.g., major), and institutional context should also be taken into account, as prior research suggests elevated depression risk among STEM students [98, 99], and greater depression and lower treatment access at public versus private institutions [100], though we did not observe associations between major and trajectory group.

### Limitations

There are some limitations to the study that can be addressed through future work. Self-report measures of depressive symptoms were only captured at two time points, limiting our ability to capture more nuanced trajectories. Additionally, this dataset was obtained before the COVID-19 pandemic, therefore it is important to consider how these results might hold if this study were to be replicated. During the pandemic, students experienced even higher rates of depression [101, 102] and post-pandemic reports of anxiety and depression are higher than pre-pandemic reports [103]. The current sample was limited by a lack of diversity, limiting our statistical power to examine the relation between sociodemographic characteristics and trajectory groups. Finally, while k-means clustering offers several analytical strengths, unlike three-step latent classification methods that explicitly account for classification uncertainty [104], k-means + + relies on selecting cluster centers and assigning observations to the nearest center, without modeling classification error, which may have introduced bias in the estimated cluster-specific effects.

Sleep-related findings should be interpreted with several limitations in mind. First, the amount of available sleep data declined over the semester and was uneven across clusters due to differences in cluster sizes. Second, to what extent might receiving biofeedback from Fitbits influence participants’ sleep behaviors? Although our study was observational, Lai et al. [105] found that while wearable-delivered interventions can improve sleep disturbance compared to control groups (e.g., usual care, sleep logs), they show limited effects on total sleep time and time in bed. Experimental studies have also demonstrated that the type of sleep-related feedback (e.g., positive vs. negative) can affect perceived sleepiness and fatigue [106], though not perceived sleep continuity [107]. While wearables enhance ecological validity, they are currently less accurate than polysomnography for measuring outcomes like total sleep time and sleep efficiency [108], although ongoing advancements in wearable technology may mitigate this limitation. As highlighted in de Zambotti et al.’s [109] state-of-the-science review, future research should continue to assess the trade-offs between ecological and clinical validity when utilizing sleep wearables. And because our sleep variable captured the longest sleep episode within a 24-hour period, future studies should distinguish between nocturnal and daytime sleep and consider curvilinear models, given the U-shaped relationship between sleep duration and depression. A final limitation is that this work was exploratory; future research may draw inferences from our results and replicate our findings in future preregistered studies [110].

## Conclusion

Despite a plethora of depressive symptom trajectory work among adolescents and emerging adults, there has been little focus on first-year college students, who are at increased risk for unfavorable depressive symptom trajectories compared to their peers in later years of college. Furthermore, there have been no studies examining associations between these trajectories with GPA or sleep outcomes using rigorous methods such as capturing continuous measures of sleep via Fitbits. Using self-reported beginning and end of spring semester depressive symptoms, k-means + + clustering yielded low-stable, increasing, decreasing, and high-stable depressive symptom trajectory groups among first-year university students. More adaptive sleep schedules and a higher spring term GPA were exhibited by the low-stable group, with one exception– the high-stable group and low-stable group shared similar sleep profiles. Lastly, we did not find any associations between sociodemographic variables and depression trajectory group membership.

Future intensive longitudinal work that combines passive sensing measures of behavior and health along with advanced statistical modeling can improve our understanding of the unique biopsychosocial mechanisms and person-level characteristics associated with trajectories of depressive symptoms. Elucidating this could inform personalized health care approaches, enable university systems to identify students who may be at risk for unfavorable mental health trajectories, and aid in the creation of just-in-time interventions. Adjusting to the first year of college can be stressful; however, future research can leverage these findings to help identify at-risk students and provide relevant interventions for promoting beneficial psychological trajectories.

## Supplementary Information

Supplementary Material 1. Descriptive statistics.

## Data Availability

The datasets used and/or analyzed during the current study are available from the corresponding author on reasonable request.

## Footnotes

1 We compared participants included in the final analyses to those excluded on baseline GPA, depressive symptoms, and sleep outcomes. Independent t-tests were used for linear variables, and Watson-Williams tests were used for circular variables (bedtime and wake time). Results showed no significant differences in fall GPA, *t*(12.72) = -0.71, *p* = 0.49 (*n* = 13 excluded), or T1 depressive symptoms, *t*(57.29) = 0.69, *p* = 0.49 (*n* = 44 excluded). Baseline sleep outcomes, assessed via the Pittsburgh Sleep Quality Index (PSQI) [70] indicated that excluded participants had a later bedtime, *F*_(1, 312)_ = 6.93, *p* = 0.01, and a shorter time in bed *t*(56.37) = -2.6, *p* = 0.01. There was no difference in TST *t*(53.79) = -1.98, *p* = 0.05, or wake time *F*_(1, 313)_ = 0.01, *p* = 0.94. The excluded group included *n* = 44 for all sleep outcomes, with one missing response each for bedtime and time in bed.

2 There were no cluster differences in baseline PSQI wake time (*F*_(3, 267)_ = 0.83, *p* = 0.48). However, there were differences in baseline PSQI TST (*F*_(3, 267)_ = 4.59, *p* = 0.004), time in bed (*F*_(3, 266)_ = 2.74, *p* = 0.04), and bedtime (*F*_(3, 266)_ = 3.20, *p* = 0.02). Compared to the low-stable group, the high-stable group had lower baseline TST (*M* = 7 H 17M, *SE* = 11 M vs. *M* = 8 H 6M, *SE* = 7 M) and time in bed (*M* = 8 H 14M, *SE* = 10 M vs. *M* = 8 H 55M, *SE* = 7 M). Pairwise comparisons found no significant differences for bedtime when comparing the increasing, decreasing, and high-stable cluster to the low-stable cluster. These sleep patterns reflected the previous month, which was winter break. Lastly, the primary monthly sleep analysis was conducted without excluding participants who had less than 20% of data available using non-transformed sleep outcomes and results remained consistent.

## References

[1] Udupa NS, Twenge JM, McAllister C, Joiner TE. Increases in poor mental health, mental distress, and depression symptoms among U.S. adults, 1993–2020. J Mood Anxiety Disorders. 2023;2:100013. 10.1016/j.xjmad.2023.100013 PMC12244020 40654375

[2] Duffy ME, Twenge JM, Joiner TE. Trends in mood and anxiety symptoms and suicide-related outcomes among U.S. undergraduates, 2007–2018: evidence from two national surveys. J Adolesc Health. 2019;65(5):590–8. 31279724 10.1016/j.jadohealth.2019.04.033

[3] Liu Y, Zhang N, Bao G, Huang Y, Ji B, Wu Y, et al. Predictors of depressive symptoms in college students: a systematic review and meta-analysis of cohort studies. J Affect Disord. 2019;244:196–208. 30352363 10.1016/j.jad.2018.10.084

[4] Denovan A, Macaskill A. An interpretative phenomenological analysis of stress and coping in first year undergraduates. Br Edu Res J. 2013;39(6):1002–24.

[5] Conley CS, Shapiro JB, Huguenel BM, Kirsch AC. Navigating the college years: developmental trajectories and gender differences in psychological functioning, cognitive-affective strategies, and social well-being. Emerg Adulthood. 2020;8(2):103–17.

[6] Bruffaerts R, Mortier P, Kiekens G, Auerbach RP, Cuijpers P, Demyttenaere K, et al. Mental health problems in college freshmen: prevalence and academic functioning. J Affect Disord. 2018;225:97–103. 28802728 10.1016/j.jad.2017.07.044 PMC5846318

[7] Schubert KO, Clark SR, Van LK, Collinson JL, Baune BT. Depressive symptom trajectories in late adolescence and early adulthood: a systematic review. Aust N Z J Psychiatry. 2017;51(5):477–99. 28415879 10.1177/0004867417700274

[8] Shore L, Toumbourou JW, Lewis AJ, Kremer P, Review. Longitudinal trajectories of child and adolescent depressive symptoms and their predictors – a systematic review and meta-analysis. Child Adoles Ment Health. 2018;23(2):107–20. 10.1111/camh.12220 32677332

[9] Musliner KL, Munk-Olsen T, Eaton WW, Zandi PP. Heterogeneity in long-term trajectories of depressive symptoms: Patterns, predictors and outcomes. J Affect Disord. 2016;192:199–211. 26745437 10.1016/j.jad.2015.12.030 PMC4761648

[10] Ames ME, Leadbeater BJ. Depressive symptom trajectories and physical health: Persistence of problems from adolescence to young adulthood. J Affect Disord. 2018;240:121–9. 30064077 10.1016/j.jad.2018.07.001

[11] Yaroslavsky I, Pettit JW, Lewinsohn PM, Seeley JR, Roberts RE. Heterogeneous trajectories of depressive symptoms: adolescent predictors and adult outcomes. J Affect Disord. 2013;148(2):391–9. 22963892 10.1016/j.jad.2012.06.028 PMC3654021

[12] Essau CA, Torre-Luque A, Lewinsohn PM, Rohde P. Patterns, predictors, and outcome of the trajectories of depressive symptoms from adolescence to adulthood. Depress Anxiety. 2020;37(6):565–75. 32526097 10.1002/da.23034

[13] Lee TK, Wickrama KAS, Kwon JA, Lorenz FO, Oshri A. Antecedents of transition patterns of depressive symptom trajectories from adolescence to young adulthood. Br J Dev Psycho. 2017;35(4):498–515. 10.1111/bjdp.12189 28707328

[14] Bulhões C, Ramos E, Severo M, Dias S, Barros H. Trajectories of depressive symptoms through adolescence and young adulthood: social and health outcomes. Eur Child Adolesc Psychiatry. 2021;30(1):65–74. 32065326 10.1007/s00787-020-01493-9

[15] Zhou S, Jin L, Liu X, Ding X, Zhu X. Developmental trajectory of depressive symptoms in Chinese college students: latent classes and gender effect. Int J Environ Res Public Health. 2022;19(6):3508. 10.3390/ijerph19063508 PMC8954229 35329200

[16] Liu Q, Tan J, Feng Z, Tu S. The role of socioeconomic status in different trajectories of depressive symptoms in Chinese college freshmen. Front Psychol. 2022;13:945959. 36033011 10.3389/fpsyg.2022.945959 PMC9412764

[17] Liu F, Yang D, Liu Y, Zhang Q, Chen S, Li W, et al. Use of latent profile analysis and k-means clustering to identify student anxiety profiles. BMC Psychiatry. 2022;22(1):12. 34986837 10.1186/s12888-021-03648-7 PMC8728926

[18] Verostek M, Miller CW, Zwickl B. Analyzing admissions metrics as predictors of graduate GPA and whether graduate GPA mediates Ph.D. completion. Phys Rev Phys Educ Res. 2021;17(2):020115.

[19] Roth PL, BeVier CA, Switzer FS III, Schippmann JS. Meta-analyzing the relationship between grades and job performance. J Appl Psychol. 1996;81(5):548–56.

[20] Pinto LH, Ramalheira DC. Perceived employability of business graduates: the effect of academic performance and extracurricular activities. J Vocat Behav. 2017;99:165–78.

[21] Awadalla S, Davies EB, Glazebrook C. A longitudinal cohort study to explore the relationship between depression, anxiety and academic performance among Emirati university students. BMC Psychiatry. 2020;20(1):448. 32917172 10.1186/s12888-020-02854-z PMC7488388

[22] Hysenbegasi A, Hass SL, Rowland CR. The impact of depression on the academic productivity of university students. J Ment Health Policy Econ. 2005;8(3):145–51. 16278502

[23] Asher BlackDeer A, Patterson Silver Wolf DA, Maguin E, Beeler-Stinn S. Depression and anxiety among college students: understanding the impact on grade average and differences in gender and ethnicity. J Am Coll Health. 2023;71(4):1091–102. 34242525 10.1080/07448481.2021.1920954

[24] Kabrita C, Hajjar-Muça T. Sex-specific sleep patterns among university students in Lebanon: impact on depression and academic performance. NSS. 2016;8:189–96. 10.2147/NSS.S104383 PMC4918802 27382345

[25] DeRoma VM, Leach JB, Leverett JP. The relationship between depression and college academic performance. Coll Student J. 2009;43(2):325–35.

[26] Creswell JD, Tumminia MJ, Price S, Sefidgar Y, Cohen S, Ren Y, et al. Nightly sleep duration predicts grade point average in the first year of college. Proc Natl Acad Sci. 2023;120(8):e2209123120. 36780521 10.1073/pnas.2209123120 PMC9974458

[27] Taylor DJ, Vatthauer KE, Bramoweth AD, Ruggero C, Roane B. The role of sleep in predicting college academic performance: is it a unique predictor? Behav Sleep Med. 2013;11(3):159–72. 23402597 10.1080/15402002.2011.602776 PMC3959895

[28] Eliasson AH, Lettieri CJ, Eliasson AH. Early to bed, early to rise! Sleep habits and academic performance in college students. Sleep Breath. 2010;14(1):71–5. 19603214 10.1007/s11325-009-0282-2

[29] Reynold AM, Bowles ER, Saxena A, Fayad R, Youngstedt SD. Negative effects of time in bed extension: a pilot study. J Sleep Med Disord. 2014;1(1):1002. 25374963 PMC4217706

[30] Supartini A, Honda T, Basri NA, Haeuchi Y, Chen S, Ichimiya A, et al. The Impact of sleep timing, sleep duration, and sleep quality on depressive symptoms and suicidal ideation amongst Japanese Freshmen: the EQUSITE study. Sleep Disorders. 2016;2016:e8737654. 10.1155/2016/8737654 PMC4794596 27042358

[31] Daghlas I, Lane JM, Saxena R, Vetter C. Genetically proxied diurnal preference, sleep timing, and risk of major depressive disorder. JAMA Psychiatry. 2021;78(8):1–8. 10.1001/jamapsychiatry.2021.0959 PMC8156187 34037671

[32] Liu L, Chen J, Liang S, Peng X, Yang W, Huang A, et al. An unusual college experience: 16-month trajectories of depressive symptoms and anxiety among Chinese new undergraduate students of 2019 during the COVID-19 pandemic. Int J Environ Res Public Health. 2023;20(6):5024. 36981933 10.3390/ijerph20065024 PMC10048813

[33] Liu X, Zhang Y, Gao W, Cao X. Developmental trajectories of depression, anxiety, and stress among college students: a piecewise growth mixture model analysis. Humanit Soc Sci Commun. 2023;10(1):736.

[34] Zhang D, Qu Y, Zhai S, Li T, Xie Y, Tao S, et al. Association between healthy sleep patterns and depressive trajectories among college students: a prospective cohort study. BMC Psychiatry. 2023;23(1):182. 36941547 10.1186/s12888-023-04596-0 PMC10026494

[35] Haghayegh S, Khoshnevis S, Smolensky MH, Diller KR, Castriotta RJ. Accuracy of wristband fitbit models in assessing sleep: systematic review and meta-analysis. J Med Internet Res. 2019;21(11):e16273. 31778122 10.2196/16273 PMC6908975

[36] Lund HG, Reider BD, Whiting AB, Prichard JR. Sleep patterns and predictors of disturbed sleep in a large population of college students. J Adolesc Health. 2010;46(2):124–32. 20113918 10.1016/j.jadohealth.2009.06.016

[37] Moo-Estrella J, Pérez-Benítez H, Solís-Rodríguez F, Arankowsky-Sandoval G. Evaluation of depressive symptoms and sleep alterations in college students. Arch Med Res. 2005;36(4):393–8. 15950081 10.1016/j.arcmed.2005.03.018

[38] American College Health Association. American College Health Association-National College Health Assessment III. Reference Group Executive Summary Spring 2024. Silver Spring: American College Health Association; 2024.

[39] Chen WL, Chen JH. Consequences of inadequate sleep during the college years: sleep deprivation, grade point average, and college graduation. Prev Med. 2019;124:23–8. 31034864 10.1016/j.ypmed.2019.04.017

[40] Li L, Wu C, Gan Y, Qu X, Lu Z. Insomnia and the risk of depression: a meta-analysis of prospective cohort studies. BMC Psychiatry. 2016;16(1):375. 27816065 10.1186/s12888-016-1075-3 PMC5097837

[41] Buysse DJ, Angst J, Gamma A, Ajdacic V, Eich D, Rössler W. Prevalence, course, and comorbidity of insomnia and depression in young adults. Sleep. 2008;31(4):473–80. 18457234 10.1093/sleep/31.4.473 PMC2279748

[42] Costello DM, Swendsen J, Rose JS, Dierker LC. Risk and protective factors associated with trajectories of depressed mood from adolescence to early adulthood. J Consult Clin Psychol. 2008;76(2):173–83. 18377115 10.1037/0022-006X.76.2.173 PMC2659847

[43] Hardy R, West H, Fisher P. Exploring attitudes towards seeking help for mental health problems among university students from racially minoritised backgrounds: a systematic review and thematic synthesis. BMC Public Health. 2025;25(1):1428. 40240964 10.1186/s12889-025-22521-w PMC12001731

[44] Freire DS, Hurd NM. Discrimination and mental health outcomes among underrepresented college students: the role of sense of belonging at predominantly white institutions. Emerg Adulthood. 2023;11(3):654–68.

[45] Chaliawala KS, Vidourek RA, King KA. Anxiety among Asian international college students in the US: a systematic literature review. J Am Coll Health. 2024;73(6):2564–71. 10.1080/07448481.2024.2317170 38442344

[46] Bokayev B. The socialization of International students in American society and its education system: a comprehensive literature review. J Social Stud Educ Res. 2024;15(3):289–316.

[47] Rockwell DM, Kimel SY. A systematic review of first-generation college students’ mental health. J Am Coll Health. 2025;73(2):519–31. 37499142 10.1080/07448481.2023.2225633

[48] Mcdossi O, Wright AL, McDaniel A, Roscigno VJ. First-generation inequality and college integration. Soc Sci Res. 2022;105:102698. 35659047 10.1016/j.ssresearch.2022.102698

[49] Ferreira D, Kostakos V, Dey AK. AWARE: mobile context instrumentation framework. Front ICT. 2015;2:6.

[50] Beloborodova P, Dutcher JM, Villalba DK, Doryab A, Creswell K, Cohen S, et al. College students’ daily mind wandering is related to lower social well-being. J Am Coll Health. 2025;73(8):2936–48. 10.1080/07448481.2024.2351417 38810254

[51] Yan R, Liu X, Dutcher JM, Tumminia MJ, Villalba D, Cohen S, et al. Identifying links between productivity and biobehavioral rhythms modeled from multimodal sensor streams: exploratory quantitative study. JMIR AI. 2024;3(1):e47194. 38875675 10.2196/47194 PMC11066747

[52] Xu X, Chikersal P, Dutcher JM, Sefidgar YS, Seo W, Tumminia MJ, et al. Leveraging collaborative-filtering for personalized behavior modeling: a case study of depression detection among college students. Proc ACM Interact Mob Wearable Ubiquitous Technol. 2021;5(1):1–27.

[53] Dutcher JM, Lederman J, Jain M, Price S, Kumar A, Villalba DK, et al. Lack of Belonging predicts depressive symptomatology in college students. Psychol Sci. 2022;33(7):1048–67. 35735353 10.1177/09567976211073135 PMC13171050

[54] Chikersal P, Doryab A, Tumminia M, Villalba DK, Dutcher JM, Liu X, et al. Detecting depression and predicting its onset using longitudinal symptoms captured by passive sensing: a machine learning approach with robust feature selection. ACM Trans Comput-Hum Interact. 2021;28(1):1–41.

[55] Doryab A, Villalba DK, Chikersal P, Dutcher JM, Tumminia M, Liu X, et al. Identifying behavioral phenotypes of loneliness and social isolation with passive sensing: statistical analysis, data mining and machine learning of smartphone and fitbit data. JMIR mHealth uHealth. 2019;7(7):e13209. 31342903 10.2196/13209 PMC6685126

[56] Radloff LS, The CES-D, Scale. A self-report depression scale for research in the general population. Appl Psychol Meas. 1977;1(3):385–401.

[57] McMullen M. How Does Fitbit Track Sleep & What is the Fitbit Sleep Score? Fitbit Enterprise. 2020. Available from: https://enterprise.fitbit.com/blog/track-sleep/. Cited 2024 Nov 14.

[58] Cui M. Introduction to the K-means clustering algorithm based on the elbow method. Account Audit Finance. 2020;1(1):5–8.

[59] Thinsungnoen T, Kaoungku N, Durongdumronchai P, Kerdprasop K, Kerdprasop N. The clustering validity with silhouette and sum of squared errors. In: The proceedings of the 2nd international conference on industrial application engineering 2015. The Institute of Industrial Applications Engineers; 2015. p. 44–51. Available from: https://www2.ia-engineers.org/conference/index.php/iciae/iciae2015/paper/view/576. Cited 2024 Jul 16.

[60] Arthur D, Vassilvitskii S. k-means++: The Advantages of Careful Seeding. Stanford, 2006.

[61] Ikotun AM, Ezugwu AE, Abualigah L, Abuhaija B, Heming J. K-means clustering algorithms: a comprehensive review, variants analysis, and advances in the era of big data. Inf Sci. 2023;622:178–210.

[62] Pewsey A, Neuhauser M, Ruxton GD. Circular Statistics in R. Oxford: Oxford University Press; 2014. p. 208.

[63] Cremers J, Klugkist I. One direction? A tutorial for circular data analysis using R with examples in cognitive psychology. Front Psychol. 2018;9:2040. 30425670 10.3389/fpsyg.2018.02040 PMC6218623

[64] Aziz NA, Anguelova GV, Marinus J, Lammers GJ, Roos RAC. Sleep and circadian rhythm alterations correlate with depression and cognitive impairment in Huntington’s disease. Parkinsonism Relat Disord. 2010;16(5):345–50. 20236854 10.1016/j.parkreldis.2010.02.009

[65] Rahman SA, Gathungu RM, Marur VR, St. Hilaire MA, Scheuermaier K, Belenky M, et al. Age-related changes in circadian regulation of the human plasma lipidome. Commun Biol. 2023;6(1):756. 37474677 10.1038/s42003-023-05102-8 PMC10359364

[66] Cambras T, Castro-Marrero J, Zaragoza MC, Díez-Noguera A, Alegre J. Circadian rhythm abnormalities and autonomic dysfunction in patients with Chronic Fatigue Syndrome/Myalgic Encephalomyelitis. PLoS ONE. 2018;13(6):e0198106. Challet E, editor. 29874259 10.1371/journal.pone.0198106 PMC5991397

[67] van Rossum G. Python reference manual. 1995. Available from: https://ir.cwi.nl/pub/5008. Cited 2024 Aug 21.

[68] R Core Team. European Environment Agency. 2020. Available from: https://www.eea.europa.eu/data-and-maps/indicators/oxygen-consuming-substances-in-rivers/r-development-core-team-2006. Cited 2024 Aug 21.

[69] RStudio Team. RStudio: Integrated Development for R. PBC, Boston: RStudio. 2020. Available from: http://www.rstudio.com.

[70] Buysse DJ, Reynolds CF, Monk TH, Berman SR, Kupfer DJ. The Pittsburgh sleep quality index: A new instrument for psychiatric practice and research. Psychiatry Res. 1989;28(2):193–213. 2748771 10.1016/0165-1781(89)90047-4

[71] Ma Z, Zhao J, Chen H, Tao Y, Zhang Y, Fan F. Temporal network of depressive symptoms across college students with distinct depressive trajectories during the COVID-19 pandemic. Depress Anxiety. 2023;2023(1):8469620. 40224588 10.1155/2023/8469620 PMC11921855

[72] Bo Shen GC, Bo J. How does change in leisure-time physical activity influence the growth trajectory of depressive symptoms in college students? J Am Coll Health. 2023;0(0):1–8. 10.1080/07448481.2023.2252503 37722822

[73] Boyraz G, Horne SG, Granda R. Depressive symptomatology and academic achievement among first-year college students: the role of effort regulation. J Coll Student Dev. 2017;58(8):1218–36.

[74] Repetto PB, Caldwell CH, Zimmerman MA. Trajectories of depressive symptoms among high risk African-American adolescents. J Adolesc Health. 2004;35(6):468–77. 15581526 10.1016/j.jadohealth.2003.12.007

[75] Salmela-Aro K, Aunola K, Nurmi JE. Trajectories of depressive symptoms during emerging adulthood: Antecedents and consequences. Eur J Dev Psychol. 2008;5(4):439–65.

[76] Hareli M. Examining the bidirectional relations between psychological functioning and academic outcomes among college students.[master’s thesis]. Chicago (IL): Loyola University Chicago; 2022.

[77] Weidman AC, Augustine AA, Murayama K, Elliot AJ. Internalizing symptomatology and academic achievement: Bi-directional prospective relations in adolescence. J Res Pers. 2015;58:106–14.

[78] Rosso AC, Wilson OWA, Papalia Z, Duffey M, Kline CE, Bopp M. Frequent restful sleep is associated with the absence of depressive symptoms and higher grade point average among college students. Sleep Health. 2020;6(5):618–22. 32247737 10.1016/j.sleh.2020.01.018

[79] Hirshkowitz M, Whiton K, Albert SM, Alessi C, Bruni O, DonCarlos L, et al. National Sleep Foundation’s sleep time duration recommendations: methodology and results summary. Sleep Health. 2015;1(1):40–3. 29073412 10.1016/j.sleh.2014.12.010

[80] American College Health Association. American College Health Association-National College Health Assessment III. Reference Group Executive Summary Spring 2025. Silver Spring: American College Health Association; 2025.

[81] Dong L, Xie Y, Zou X. Association between sleep duration and depression in US adults: a cross-sectional study. J Affect Disord. 2022;296:183–8. 34607059 10.1016/j.jad.2021.09.075

[82] Brooks PR, Girgenti AA, Mills MJ. Sleep patterns and symptoms of depression in college students. Coll Student J. 2009;43(2):464–73.

[83] Pilcher JJ, Rummel EG, Lamm C. Sleep quality, sleep quantity, and sleep timing: contrasts in Austrian and U.S. college students. Front Sleep. 2024;3:1487739. 41424489 10.3389/frsle.2024.1487739 PMC12713796

[84] Okano K, Kaczmarzyk JR, Dave N, Gabrieli JDE, Grossman JC. Sleep quality, duration, and consistency are associated with better academic performance in college students. NPJ Sci Learn. 2019;4:16. 31583118 10.1038/s41539-019-0055-z PMC6773696

[85] Sletten TL, Weaver MD, Foster RG, Gozal D, Klerman EB, Rajaratnam SMW, et al. The importance of sleep regularity: a consensus statement of the National Sleep Foundation sleep timing and variability panel. Sleep Health. 2023;9(6):801–20. 37684151 10.1016/j.sleh.2023.07.016

[86] Windred DP, Burns AC, Lane JM, Saxena R, Rutter MK, Cain SW, et al. Sleep regularity is a stronger predictor of mortality risk than sleep duration: a prospective cohort study. Sleep. 2024;47(1):zsad253. 37738616 10.1093/sleep/zsad253 PMC10782501

[87] Sleep | Cornell Health. Available from: https://health.cornell.edu/resources/health-topics/sleep#. Cited 2024 Aug 26.

[88] House LA, Neal C, Kolb J. Supporting the mental health needs of first generation college students. J Coll Student Psychother. 2020;34(2):157–67.

[89] Jenkins SR, Belanger A, Connally ML, Boals A, Durón KM. First-generation undergraduate students’ social support, depression, and life satisfaction. J Coll Couns. 2013;16(2):129–42.

[90] Noel JK, Lakhan HA, Sammartino CJ, Rosenthal SR. Depressive and anxiety symptoms in first generation college students. J Am Coll Health. 2023;71(6):1906–15. 34314656 10.1080/07448481.2021.1950727

[91] Larcombe W, Ryan T, Baik C. Are international students relatively resilient? Comparing international and domestic students’ levels of self-compassion, mental health and wellbeing. High Educ Res Dev. 2024;43(2):362–76.

[92] Hirai R, Frazier P, Syed M. Psychological and sociocultural adjustment of first-year international students: trajectories and predictors. J Couns Psychol. 2015;62(3):438–52. 10.1037/cou0000085 25961754

[93] Cheng S, An D, Yao Z, Liu JJW, Ning X, Wong JPH, et al. Association between mental health knowledge level and depressive symptoms among Chinese college students. Int J Environ Res Public Health. 2021;18(4):1850. 33672872 10.3390/ijerph18041850 PMC7918134

[94] Chen JA, Stevens C, Wong SHM, Liu CH. Psychiatric symptoms and diagnoses among U.S. college students: a comparison by race and ethnicity. Psychiatr Serv. 2019;70(6):442–9. 30914001 10.1176/appi.ps.201800388 PMC6628693

[95] Zhou J, Qu J, Ji S, Bu Y, Hu Y, Sun H, et al. Research trends in college students’ sleep from 2012 to 2021: a bibliometric analysis. Front Psychiatry. 2022;13:1005459. 36203831 10.3389/fpsyt.2022.1005459 PMC9530190

[96] Zhou X, Zhou AQ, Sun X. Prevalence of common mental concerns and service utilization among international students studying in the U.S. Counselling Psychol Q. 2022;35(3):483–502.

[97] Lippincott JA, German N. From blue collar to Ivory Tower: counseling first-generation, working-class students. Special populations in college counseling: a handbook for mental health professionals. Alexandria: American Counseling Association; 2007. p. 89–98.

[98] Busch CA, Mohammed TF, Nadile EM, Cooper KM. Aspects of online college science courses that alleviate and exacerbate undergraduate depression. Mittal P, editor. PLoS ONE. 2022;17(6):e0269201. 10.1371/journal.pone.0269201 PMC9159593 35648764

[99] Reidy DE, Wood L. The mental health of undergraduate women majoring in STEM. J Am Coll Health. 2025;73(3):914–9. 38227921 10.1080/07448481.2023.2299426

[100] Ketchen Lipson S, Gaddis SM, Heinze J, Beck K, Eisenberg D. Variations in student mental health and treatment utilization across US colleges and universities. J Am Coll Health. 2015;63(6):388–96. 25942473 10.1080/07448481.2015.1040411

[101] Sharaievska I, McAnirlin O, Browning MHEM, Larson LR, Mullenbach L, Rigolon A, et al. Messy transitions: Students’ perspectives on the impacts of the COVID-19 pandemic on higher education. High Educ (Dordr). 2022:1–8. 10.1007/s10734-022-00843-7 PMC9020423 35463941

[102] Woolston C. Signs of depression and anxiety soar among US graduate students during pandemic. Nature. 2020;585(7823):147–8. 32811983 10.1038/d41586-020-02439-6

[103] Nails JG, Maffly-Kipp J, DeShong HL, Lowmaster SE, Kurtz JE. A crisis in college student mental health? Self-ratings of psychopathology before and after the COVID-19 pandemic. Psychol Assess. 2023;35(11):1010–8. 37289503 10.1037/pas0001241

[104] Vermunt JK. Latent class modeling with covariates: two improved three-step approaches. Polit anal. 2010;18(4):450–69.

[105] Lai MYC, Mong MSA, Cheng LJ, Lau Y. The effect of wearable-delivered sleep interventions on sleep outcomes among adults: a systematic review and meta-analysis of randomized controlled trials. Nurs Health Sci. 2023;25(1):44–62. 36572659 10.1111/nhs.13011

[106] Gavriloff D, Sheaves B, Juss A, Espie CA, Miller CB, Kyle SD. Sham sleep feedback delivered via actigraphy biases daytime symptom reports in people with insomnia: implications for insomnia disorder and wearable devices. J Sleep Res. 2018;27(6):e12726. 29989248 10.1111/jsr.12726

[107] Robson AR, Ellis JG, Elder GJ. Poor false sleep feedback does not affect pre-sleep cognitive arousal or subjective sleep continuity in healthy sleepers: a pilot study. Sleep Biol Rhythms. 2022;20(4):467–72. 38468629 10.1007/s41105-022-00390-9 PMC10899903

[108] Schyvens AM, Peters B, Van Oost NC, Aerts JM, Masci F, Neven A, et al. A performance validation of six commercial wrist-worn wearable sleep-tracking devices for sleep stage scoring compared to polysomnography. Sleep Adv. 2025;6(2):zpaf021. 40303381 10.1093/sleepadvances/zpaf021 PMC12038347

[109] de Zambotti M, Goldstein C, Cook J, Menghini L, Altini M, Cheng P, et al. State of the science and recommendations for using wearable technology in sleep and circadian research. Sleep. 2024;47(4):zsad325. 38149978 10.1093/sleep/zsad325

[110] Matsushita M, Koyama A, Ushijima H, Mikami A, Katsumata Y, Kikuchi Y, et al. Sleep duration and its association with sleepiness and depression in *ronin*-*sei* preparatory school students. Asian J Psychiatry. 2014;9:61–6. 10.1016/j.ajp.2014.01.006 24813039

