# Supplementary material for: Depression trajectories and their associations with academic performance and sleep patterns among first-year college students
Source: BMC Psychol. 2026 May 2;14:1173. doi: 10.1186/s40359-026-04404-w (PMC13463478; doi:10.1186/s40359-026-04404-w)
Supplement: Supplementary file 1 [file 40359_2026_4404_MOESM1_ESM.docx]

**Supplementary Materials**

**Table S1.** Sample sizes for sleep outcomes across months.

| Time | Total | Low-stable | Decreasing | Increasing | High-stable |
| --- | --- | --- | --- | --- | --- |
| Month 1 | 208 | *n =* 84 | *n =* 45 | *n =* 52 | *n =* 27 |
| Month 2 | 200 | *n =* 81 | *n =* 44 | *n =* 50 | *n =* 25 |
| Month 3 | 177 | *n =* 69 | *n =* 40 | *n =* 44 | *n =* 24 |
| Month 4 | 168 | *n =* 72 | *n =* 39 | *n =* 37 | *n =* 20 |

Note: The range of observations was 5-28 for month 1 (*M* = 24.89), month 2 (*M* = 23.19), month 3 (*M* = 22.63), and 5-27 observations for month 4 (*M* = 19.90).

***Table S2****. Skewness and kurtosis values for linear sleep outcomes.*

|  |  |  | Total sleep time | | Time in bed | |
| --- | --- | --- | --- | --- | --- | --- |
| Month | Cluster | *n* | Skewness | Kurtosis | Skewness | Kurtosis |
| 1 | Low | 2084 | -0.49 | 3.99 | -0.52 | 4.18 |
| 1 | Decreasing | 1163 | -0.29 | 3.54 | -0.30 | 3.43 |
| 1 | Increasing | 1290 | -0.32 | 3.78 | -0.32 | 3.80 |
| 1 | High | 641 | -0.53 | 4.00 | -0.57 | 4.18 |
| 2 | Low | 1847 | -0.37 | 3.81 | -0.39 | 3.85 |
| 2 | Decreasing | 1075 | -0.07 | 3.44 | -0.07 | 3.40 |
| 2 | Increasing | 1137 | -0.19 | 3.38 | -0.21 | 3.50 |
| 2 | High | 579 | -0.22 | 3.43 | -0.23 | 3.50 |
| 3 | Low | 1548 | -0.47 | 3.95 | -0.48 | 4.08 |
| 3 | Decreasing | 967 | -0.14 | 3.62 | -0.16 | 3.49 |
| 3 | Increasing | 981 | -0.21 | 3.36 | -0.28 | 3.32 |
| 3 | High | 509 | -0.14 | 3.57 | -0.17 | 3.62 |
| 4 | Low | 1365 | -0.46 | 3.86 | -0.46 | 3.93 |
| 4 | Decreasing | 812 | -0.17 | 3.54 | -0.19 | 3.51 |
| 4 | Increasing | 771 | -0.34 | 3.46 | -0.37 | 3.52 |
| 4 | High | 396 | -0.03 | 5.83 | -0.07 | 5.86 |

Note: Outliers were detected using the interquartile (IQR) method and winsorized. Analyses were repeated using winsorized values of total sleep time and time in bed and results remained consistent.

**Table S3.** Descriptive statistics for circular sleep outcomes.

|  |  |  | Bedtime | | | Wake time | | |
| --- | --- | --- | --- | --- | --- | --- | --- | --- |
| Month | Cluster | *n* | Mean Direction (radians) | Mean Resultant Length (ρ) | Circular SD (radians) | Mean Direction (radians) | Mean Resultant Length (ρ) | Circular SD (radians) |
| 1 | Low | 2084 | 2.04 | 0.89 | 0.48 | 1.29 | 0.89 | 0.47 |
| 1 | Decreasing | 1163 | 2.17 | 0.89 | 0.49 | 1.31 | 0.89 | 0.48 |
| 1 | Increasing | 1290 | 2.17 | 0.89 | 0.49 | 1.38 | 0.88 | 0.50 |
| 1 | High | 641 | 2.07 | 0.83 | 0.61 | 1.29 | 0.85 | 0.57 |
| 2 | Low | 1847 | 2.12 | 0.86 | 0.56 | 1.34 | 0.87 | 0.53 |
| 2 | Decreasing | 1075 | 2.22 | 0.87 | 0.52 | 1.37 | 0.86 | 0.56 |
| 2 | Increasing | 1137 | 2.25 | 0.84 | 0.58 | 1.45 | 0.85 | 0.58 |
| 2 | High | 579 | 2.09 | 0.78 | 0.70 | 1.29 | 0.79 | 0.68 |
| 3 | Low | 1548 | 2.11 | 0.85 | 0.58 | 1.32 | 0.86 | 0.55 |
| 3 | Decreasing | 967 | 2.25 | 0.85 | 0.56 | 1.38 | 0.86 | 0.56 |
| 3 | Increasing | 981 | 2.24 | 0.82 | 0.64 | 1.42 | 0.81 | 0.64 |
| 3 | High | 509 | 2.11 | 0.78 | 0.70 | 1.30 | 0.80 | 0.66 |
| 4 | Low | 1365 | 2.12 | 0.83 | 0.61 | 1.38 | 0.83 | 0.62 |
| 4 | Decreasing | 812 | 2.22 | 0.81 | 0.65 | 1.40 | 0.81 | 0.65 |
| 4 | Increasing | 771 | 2.27 | 0.80 | 0.66 | 1.52 | 0.81 | 0.64 |
| 4 | High | 396 | 2.02 | 0.77 | 0.73 | 1.25 | 0.77 | 0.73 |

Note: See Table 4-5 in the main manuscript for mean direction and circular standard deviation values in timestamp format. Higher rho (ρ) values indicates that the data cluster around a central direction and thus are appropriate for Watson-Williams testing (Pewsey et al., 2013). Rayleigh tests were also conducted to confirm that the data was not uniform (all p’s <.05) (Landler et al., 2018), thus meeting the Watson-William assumptions.

***Table S4.*** *Concentration parameter of von Mises distribution.*

| Month | Cluster | Bedtime κ | Wake time κ |
| --- | --- | --- | --- |
| 1 | Low | 4.88 | 4.98 |
| 1 | Decreasing | 4.70 | 4.83 |
| 1 | Increasing | 4.65 | 4.56 |
| 1 | High | 3.29 | 3.69 |
| 2 | Low | 3.77 | 4.15 |
| 2 | Decreasing | 4.28 | 3.76 |
| 2 | Increasing | 3.53 | 3.59 |
| 2 | High | 2.67 | 2.79 |
| 3 | Low | 3.59 | 3.83 |
| 3 | Decreasing | 3.71 | 3.77 |
| 3 | Increasing | 3.08 | 3.04 |
| 3 | High | 2.69 | 2.92 |
| 4 | Low | 3.27 | 3.23 |
| 4 | Decreasing | 3.00 | 2.97 |
| 4 | Increasing | 2.92 | 3.06 |
| 4 | High | 2.52 | 2.52 |

Note: When running the Watson-William test, RStudio gave a warning that the concentration parameters were not equal between groups (one of the model assumptions for Watson-Williams test).

***Table S5****. Percentage of data falling within sleep windows.*

|  |  | Bedtime | | | | Wake time | | | |
| --- | --- | --- | --- | --- | --- | --- | --- | --- | --- |
| Time | Cluster | 10:00 -11:59PM | 12:00 -1:59AM | 2:00 -3:59AM | Other | 6:00 -7:59AM | 8:00 -9:59AM | 10:00 -11:59AM | Other |
| Month 1 | Low | 11.9 | 43.5 | 33.6 | 10.9 | 20.7 | 51.8 | 17.7 | 9.8 |
| Month 1 | Decreasing | 6.1 | 38.5 | 37.7 | 17.6 | 22.4 | 48.5 | 18.4 | 10.7 |
| Month 1 | Increasing | 5.9 | 37.2 | 41.2 | 15.7 | 20.4 | 45.2 | 23.3 | 11.1 |
| Month 1 | High | 12.0 | 41.3 | 28.9 | 17.8 | 27.0 | 46.0 | 14.5 | 12.5 |
| Month 2 | Low | 8.6 | 40.5 | 33.6 | 17.3 | 18.6 | 49.9 | 20.8 | 10.6 |
| Month 2 | Decreasing | 6.3 | 34.0 | 39.3 | 20.3 | 20.4 | 43.8 | 22.0 | 13.9 |
| Month 2 | Increasing | 7.5 | 28.2 | 41.2 | 23.1 | 16.7 | 41.8 | 25.8 | 15.7 |
| Month 2 | High | 13.5 | 34.4 | 30.7 | 21.4 | 26.3 | 41.1 | 15.9 | 16.8 |
| Month 3 | Low | 9.6 | 41.5 | 33.2 | 15.7 | 21.1 | 49.1 | 18.3 | 11.5 |
| Month 3 | Decreasing | 5.2 | 32.7 | 39.1 | 23.1 | 19.8 | 44.0 | 21.5 | 14.8 |
| Month 3 | Increasing | 7.1 | 30.3 | 39.0 | 23.5 | 17.4 | 41.0 | 24.0 | 17.6 |
| Month 3 | High | 12.2 | 32.2 | 32.8 | 22.8 | 23.4 | 41.8 | 17.3 | 17.5 |
| Month 4 | Low | 8.5 | 38.1 | 34.7 | 18.7 | 17.1 | 43.8 | 24.6 | 14.5 |
| Month 4 | Decreasing | 8.7 | 30.9 | 34.6 | 25.7 | 20.2 | 36.8 | 24.9 | 18.1 |
| Month 4 | Increasing | 5.8 | 28.7 | 37.9 | 27.6 | 15.0 | 37.1 | 29.6 | 18.3 |
| Month 4 | High | 15.7 | 36.4 | 26.5 | 21.5 | 23.0 | 38.1 | 20.2 | 18.7 |

***Figure S1.*** *Cluster validation (elbow method).*

***Figure S2.*** *Cluster validation (silhouette method).*

*Note:* We manually calculated the average silhouette width, revealing that *k* = 2 had a width of 0.441, *k* = 3 had a width of 0.416, and *k* = 4 had a width of 0.422. Given that the elbow method suggested *k* = 4 and there was empirical evidence supporting the existence of four unique depressive symptom trajectory groups, we set *k* = 4.

**References**

Landler L, Ruxton GD, Malkemper EP. Circular data in biology: advice for effectively

implementing statistical procedures. Behavioral ecology and sociobiology. 2018 Aug;72:1-0.

Pewsey A, Neuhäuser M, Ruxton GD. Circular statistics in R. OUP Oxford; 2013 Sep 26.
